# Catching the news: Processing strategies in listening to dialogs as measured by ERPs

**DOI:** 10.1186/1744-9081-3-53

**Published:** 2007-10-08

**Authors:** Ulrike Toepel, Ann Pannekamp, Kai Alter

**Affiliations:** 1 Neuropsychology and Neurorehabilitation Service, University Hospital and University of Lausanne, Switzerland; 2 Institute of Psychology, Humboldt University Berlin, Germany; 3 Institute of Neuroscience, Newcastle Auditory Group, University of Newcastle, UK

## Abstract

**Background:**

The online segmentation of spoken single sentences has repeatedly been associated with a particular event-related brain potential. The brain response could be attributed to the perception of major prosodic boundaries, and was termed Closure Positive Shift (CPS). However, verbal exchange between humans is mostly realized in the form of cooperative dialogs instead of loose strings of single sentences. The present study investigated whether listeners use prosodic cues for structuring larger contextually embedded utterances (i.e. dialogs) like in single sentence processing.

**Methods:**

ERPs were recorded from listeners (n = 22) when presented with question-answer dialogs in German. The prosody of the answer (target sentence) either matched the context provided by a question or did not match the context question.

**Results:**

CPS responses to the processing of the target sentences are elicited, first, when listeners encounter information comprising 'novelties', i.e. information not mentioned in the preceding question but facts corrected between context and target. Thereby it is irrelevant whether the actual prosody of the target sentence is in congruence with the informative status or not. Second, when listeners encounter target sentences which do not convey any novelties but only previously 'given' already known information, the structuring of the speech input is driven by prosody again. The CPS is then elicited when listeners perceive major prosodic boundaries similar as for the processing of context-free single sentences.

**Conclusion:**

The study establishes a link between the on-line structuring of context-free (single sentences) and context-embedded utterances (dialogs) as measured by ERPs. Moreover, the impact of prosodic phrasing and accentuation on the perception of spoken utterances on and beyond sentence level is discussed.

## Background

Humans use dialog conversation constantly to exchange information between them. We talk to each other at home, at work or on the phone with the goal to communicate information which we believe to be new or important for others. Most people never recognize that the verbal exchanges between them are arranged in a highly structured way. Yet, when people talk, they usually connect their current utterances with preceding expressions of themselves or others. The philologist Hermann Paul noticed this inherent connection between questions that people ask, and the tonal realization of subsequent answers already in the 19^th ^century [1].

Nowadays, the term 'information structure' is used to designate that connected utterances are composed of so-called 'information units' [2] which can be larger than a single syllable or word. Using a simplified view, these units can either consist of 'novelties' or can comprise previously 'given', thus known, facts. In general, the term 'focus' is used to refer to information centers which are currently novel for listeners or contrast with previous assertions of dialog partners (interlocutors). On the contrary, information which listeners already encountered earlier in a discourse is referred to as non-focused or given information.

The proportions of focused and non-focused information within a discourse are subject to constant dynamics. While interlocutors speak about a certain theme they take alternating turns as speakers and listeners. These alternating turns force interlocutors to persistently reconsider which part of information is already shared between them and their conversation partners (common knowledge) and which part conveys novelties and/or contrastive assertions. Shared information between interlocutors can then provide the context for forthcoming utterances [3]. However, when information is not yet shared it must somehow be highlighted or focused, respectively, to enter the conversational common ground of the interlocutors.

German, as the language under investigation here, provides several linguistic opportunities to realize an information focus in written and/or spoken language (focus position = **bold word**). These can first be syntactic means accompanied by word order changes (Jeff likes **chocolate**. → It is **chocolate **that Jeff likes.) Moreover, an information focus can be induced by semantic-pragmatic requirements, e.g. by wh-words (Who likes chocolate? → **Jeff **/What does Jeff like? → **Chocolate**). In spoken language only, a focus can also be overtly highlighted by prosody or accentuation, respectively (Jeff likes **chocolate**.).

The study at hand serves to explore the interplay of semantic-pragmatic and prosodic factors (i.e. accentuation) in processing the focused information in dialogs. Event-related potentials (ERPs) were utilized to particularly investigate on-line interactions between the semantic-pragmatic and the prosodic focusing device. For this purpose, the electrophysiological consequences of perceiving matching and non-matching associations of pragmatic focus and the (prosodic) focus accentuation during spoken dialog comprehension were compared (see Table 1).

**Table 1:** Examples of the dialogs with a focus on 'Anna' in the target sentence (F3) or non-focused given information (G3).

***Condition GG***	***Condition FF***
G1: Am Samstag hat Peter mir etwas versprochen. *Peter promised me something on Saturday.*	F1: Am Samstag hat Peter mir etwas versprochen. *Peter promised me something on Saturday.*
G2: Hat er dir versprochen, Anna zu entlasten? *Did he promise you to support Anna?*	F2: Hat er dir versprochen, Frauke zu entlasten? *Did he promise you to support Frauke?*
G3: Er hat mir versprochen, [Anna]_G _zu entlasten und die Küche zu putzen. *He promised me to support Anna* *and to clean the kitchen.*	F3: Er hat mir versprochen, [ANNA]_F _zu entlasten und die Küche zu putzen. *He promised me to support Anna* *and to clean the kitchen.*

***Condition FG***	***Condition GF***

F1: Am Samstag hat Peter mir etwas versprochen.	G1: Am Samstag hat Peter mir etwas versprochen.
F2: Hat er dir versprochen, Frauke zu entlasten?	G2: Hat er dir versprochen, Anna zu entlasten?
G3: Er hat mir versprochen, [Anna]_G _zu entlasten und die Küche zu putzen.	F3: Er hat mir versprochen, [ANNA]_F _zu entlasten und die Küche zu putzen.

Condition GG = NO FOCUS or GIVEN + appropriate accentuation

Condition FF = FOCUS+ appropriate accentuation

Condition FG = FOCUS + inappropriate accentuation

Condition GF = NO FOCUS or GIVEN + inappropriate accentuation

Appropriate associations of context and target are signaled by identical letters (G1+G2+G3 = condition GG and F1+F2+F3 = condition FF). Inappropriate associations of contextual information and target sentences were created by combining the context of one condition and the target sentence of the opposite condition (G1+G2+F3 = condition GF or F1+F2+G3 = condition FG). Capitalized words signal focus accents.

For reasons of intelligibility, we will henceforth refer to the contextually driven pragmatic information centers just as 'focus'. The actual prosodic realization of these information centers will be referred to as '(focus) accentuation'. Yet, a focus does not only bear consequences for the accentuation of the information center. Rather, words preceding or following the focus position or information center, respectively, are also influenced in their prosodic properties, i.e. to enhance the prominence of the focus with respect to surrounding sentence elements ([4] for a longer linguistics-based discussion).

Prior studies aiming at behavioral responses during dialog processing have shown that semantic-pragmatically focused information is recognized faster and easier when it is accented [5,6]. Moreover, focused information which is not accented is hardly acceptable for listeners while the superfluous accentuation of non-focused information is more readily accepted [7,8]. In single sentence processing, the influence of accent positions on sentence interpretation has been studied as well. Yet, a study by Price, Ostendorf, Shattuck-Hufnagel and Fong [9] reported only a minor influence of accent positions on the disambiguation of syntactic structures. However, the study revealed a substantial influence of the positions of major prosodic phrase boundaries on syntactic disambiguation. On the other hand, two other studies do also report robust effects of accent positions on the syntactic disambiguation of sentences [10,11].

With respect to ERP responses to the processing of prosodic and pragmatic information, findings are not straightforward. In single sentence processing, a positive-going ERP is often found when listeners perceive major prosodic boundaries [12-15]. Major prosodic boundaries signal the closure of intonational phrases within sentences. These boundaries manifest in tonal movements on the last syllables preceding the edges, a lengthening of the prefinal boundary syllable and an optional pause [16]. The ERP deflections to these boundaries display a latency of approx. 500 ms, and a centro-parietal scalp distribution. Due to their eliciting factors (i.e. major prosodic phrase boundaries) the ERP has been termed Closure Positive Shift (CPS), and is interpreted as an on-line marker for speech segmentation.

However, when listeners process utterances beyond single sentences (i.e. in dialogs), the CPS reveals diverging elicitation factors. Hruska and coworkers [17,18] conducted a study on the processing of dialogues in German. They presented listeners with context questions either comprising the wh-pronoun 'who' or 'what'. The 'who'-question induced a novelty focus on a noun while the 'what'-question gave rise to a focus on a verb in a target sentence. In order to determine whether the elicitation of the CPS predominantly relies on the pragmatic aspects of the dialog (i.e. the contextually assigned focus positions) or on the actual prosodic realization (i.e. the focus accentuation), Hruska et al. included an additional manipulation in their design. The questions including the pronoun 'who' (inducing a noun focus) were either followed by target sentences comprising the matching (noun) or the non-matching (verb) accentuation. In addition, questions including the pronoun 'what' (inducing a verb focus) were either followed by the matching (verb) or the non-matching (noun) accentuation.

Most importantly, the results show that when listeners are presented with contextually embedded sentences (i.e. dialogs) the CPS is not elicited by perceiving major prosodic boundaries as during context-free single sentence processing [12]. When the context-induced focus position and the accent position in the target sentence are identical, the CPS was elicited to this focused and accented position ('who'-question → CPS to the noun; 'what'-question → CPS to the verb). Yet, when focus and accent position were incongruent the ERP outcomes were less clear-cut. When a 'what'-question (inducing a verb focus) was followed by a target sentence with noun accentuation, a CPS was elicited in accordance with the accent position (i.e. the noun) which was not the focus position. Moreover, the missing accent in the focus position (i.e. verb) elicited an N400. In contrast, the association of the 'who'-question (inducing a noun focus) with a target sentence conveying a verb accent induces a CPS and a biphasic N400-P600 pattern in correspondence to the focused noun which was not accented. Thus, the results of Hruska et al. are not unequivocal in determining whether the CPS in dialogs indexes the perception of a contextually promoted focus or of a focus accent or both.

The N400 effects which were consistently caused by missing accents on focused sentence constituents are proposed to reflect semantic integration difficulties. In particular, they were attributed to the expectation of accents by listeners when encountering a focus position which was not marked by accentuation means. Moreover, the occurrence of a P600 is suggested to signal the revision of a dialog's information structure due to inconsistencies between the pragmatic (focus) and the prosodic structure (i.e. accentuation). Critically, the data of the study are only displayed and statistically evaluated from the absolute sentence onsets. Although the additionally provided acoustic analyses allow for a loose mapping of the critical sentence positions (i.e. noun and verb) with the ERP effects, the exact time course of the evoked responses are not unambiguous.

The processing of focused information in the visual domain has also been found to yield a positive-going ERP. Bornkessel, Schlesewsky and Friederici [19] employed word order scrambling which resulted in the syntactic focus positions. The perception of these focused elements elicited a posterior parietal positivity with a latency of 280–480 ms. The ERP was then termed 'focus positivity'. Yet, the evoking conditions, latency, and scalp distribution of the visual 'focus positivity' are similar to the CPS found in auditory dialog processing [17,18]. As written language does not convey overt prosodic features, the data provide a hint as to the independence of the positive-going 'focus CPS' from the actual accentuation of a focus.

With respect to the electrophysiological consequences of inadequate accentuation various effects have been previously reported. Heim and Alter [20] report a frontal P200 to unexpected sentence-initial and an N400 for sentence-medial accents in German. Further, Mietz, Toepel, Ischebeck and Alter [21] discussed a still earlier appearing centro-parietal negativity (EN) peaking at 120 ms to unexpected sentence-medial accentuation in German. For Japanese, Ito and Garnsey [22] find a posterior positivity between 250–500 ms for missing sentence-initial focus accents but a later fronto-temporal negativity for missing sentence-medial accents. Furthermore, Magne et al. [23] discuss a sustained centro-posterior positivity between 300–1000 ms for 'pop-out' accents in medial and final sentence positions in French.

Up to date, the ERP data on the impact of pragmatic and prosodic aspects on utterance processing at and beyond single sentence level are still far from consistent. Yet, a line of ERP research concerned with intra- and extrasentential context effects on semantic processing as reflected in particular by the N400 component reveals major compliance between both kinds of contextual influences [24,25]. Evidence from the N400 component indicates that the processing of intra-sentential contextual requirements and extra-sentential semantic preconditions (e.g. constraints on semantic interpretation introduced by a preceding context or by world knowledge) are effective at a comparable speed and strength, and possibly subserved by identical neural networks [26].

The current study thus aims at determining the eliciting factors of the CPS in the context-bound processing of dialogs. Furthermore, we will explore on potential influences of inappropriate prosodies on dialog perception. In particular, a link between the CPS as an on-line marker for utterance segmentation in context-free (i.e. single sentences → CPS at major prosodic boundaries) vs. context-bound (i.e. dialogs → CPS to focus/accent positions) speech processing is to be drawn.

In line with prior research [17,18], we propose that the online speech segmentation processes for context-free and context-embedded utterances manifests in a similar ERP component, namely in the CPS. Yet, the events which elicit the CPS in sentences and dialogs seem to differ. We suggest that this difference arises from a rather eclectic and economic strategy of listeners to use the most relevant cues for utterance structuring (leaving aside here the interpretation-indispensable lower-level phonological, semantic and syntactic cues). In single sentences, speech segmentation by means of prosodic boundaries can help to prevent misunderstandings as in the sentence 'When you learn gradually you worry more.' [9]. In larger discourse and dialogs, however, prosodic boundaries are not as informative as in single sentences [27,28]. In lieu of recognizing the syntax of an utterance, it is superior to determine its informational content, i.e. the information centers. As mentioned beforehand, these information foci can be indicated by context-driven semantic-pragmatic means as well as by accentuation.

We created three-sentence dialogs to explore on the interplay of pragmatic and prosodic factors in discourse processing (see Table 1). In particular, dialogs were constructed in which the last (target) sentence either comprised a 'novelty' expressed by the corrected assumption of the interlocutor in noun position (i.e. focused information; *condition FF*) or only previously mentioned 'given' information (i.e. non-focused; *condition GG*). Since the dialogs were spoken in a collaborative setting between two speakers, the dialogs were naturally accompanied by a corresponding focus or no-focus accentuation (see section on prosodic properties for details).

We propose in general that the conversational contexts (i.e. questions posed by speakers and their prosody) influence listeners' expectations on a focus position in the target utterance. We predict that when contextual cues indicate a focus, listeners then use the anticipated focus position to structure the dialog. In turn, when utterances bear a noun focus with its corresponding accentuation (*condition FF*), the CPS should be elicited in convergence to this focused and accented noun position. When the dialog does, however, not point to the existence of a focus (*condition GG*) listeners are expected to structure the target utterance by means of the internal major prosodic boundaries. The CPS should then be apparent when listeners perceive the major prosodic boundaries as shown for the perception of context-free single sentences [17].

In addition and to specifically disentangle contextually-pragmatically and prosodically driven ERP effects in dialog perception, a further manipulation entered the study design. First, we combined the contexts which give rise to a 'focus' position in the target sentence with the prosodic realization of non-focused 'given' information (*condition FG*). Second, the contexts which render all information in the target sentence as non-focused 'given' were combined with the target sentences incorporating the accentuation of a 'focus' (*condition GF*).

As mentioned beforehand, prior ERP data could not unequivocally determine whether information structural conflicts are resolved by listeners in favor of the contextually triggered pragmatic focus structure or the actual accentuation. In turn, our hypotheses on the perceptual outcomes of such a conflict have to be two-fold.

On the one hand, if listeners process the dialogs with an inadequate accentuation by primarily regarding contextual-pragmatic cues, a CPS would be expected to the target sentence noun if this bears a focus (*condition FG*). Thus, the latencies of the CPS in condition FG and FF would be congruous then since the target sentences of both conditions are preceded by the same contextual information. In the opposite case, where the target sentence noun only conveys non-focused given information (*condition GF*) a CPS would be expected at the noun-preceding major prosodic phrase boundary due to the lack of a focus position. If this assumption is valid, the CPS timing between the context-identical conditions GF and GG should be similar.

On the other hand, if listeners structure the dialog targets conveying inappropriate accentuation patterns by predominantly relying on the misleading prosody, a CPS should be induced by the noun focus accent in condition GF. The CPS latencies between condition GF and FF would then be coinciding since the target sentences of both conditions bear the same accentuation pattern. Yet, when the target sentences bear the prosody of non-focused given information (*condition FG*) listeners should exhibit a CPS to the perception of the noun-preceding major prosodic boundary due to the absence of a (focus) accent. Thus, the CPS pattern should then be similar between conditions FG and GG since both conditions convey prosodically identical target sentences.

## Methods

### Participants

Twenty-two volunteers (11 female) took part in the experiment. Their mean age was 24.7 y (sd 3.21). All were right-handed according to the Edinburgh Handedness Inventory [29], and without any known neurological or hearing disorders.

### Stimulus materials

The four dialog conditions consisted of three sentences each (see Table 1). The introductory sentences (F1 and G1) were identical between conditions. The interplay between the context question (F2 or G2) and the target sentence (F3 and G3) determines the particular information structure of a dialog. Question F2 establishes a contrast between the noun 'Frauke' and the noun 'Anna' in the target sentence F3. The interplay between F2 and F3 gives rise to a (correction) focus in the position of the noun 'Anna' (*condition FF*). In *condition GG*, the sentences G2 and G3 both convey the noun 'Anna'. Thus, no contrast between G2 and G3 is established. The target sentence G3 does not comprise a focus in noun position but only information that is already contextually given or non-focused, respectively.

Forty-four dialogs were created for either condition (*conditions FF and GG*). The target sentences were syntactically identical, and consisted of the same number of constituents. They were produced by two female speakers of Standard German mimicking a quasi-natural dialog situation. In turn, the pragmatic structure (focus or no focus on the noun) and the accentuation (focus accentuation or prosody of given information) match each other in the conditions FF and GG. The recordings took place in a sound-attenuated room, and were then digitized as individual sound files (44.1 kHz, Mono, 16 bit). The loudness of the recordings was adapted.

In addition to these conditions with matching associations of the contextual-pragmatic information and accentuation patterns, two conditions with non-matching pairings were created. For this purpose, the sentences F1 and F2 were combined with G3 (*condition FG*); and the sentences G1 and G2 were combined with F3 (*condition GF*). The interplay between context and target sentence in condition FG determines a focus on the noun 'Anna'. However, the accentuation of sentence G3 is the one of non-focused given information. In contrast, the interplay of context and target in condition GF renders all information in sentence F3 non-focused or given. However, sentence F3 conveys the accentuation of a focus.

### Prosodic properties of the dialog materials

**Figure 1:**
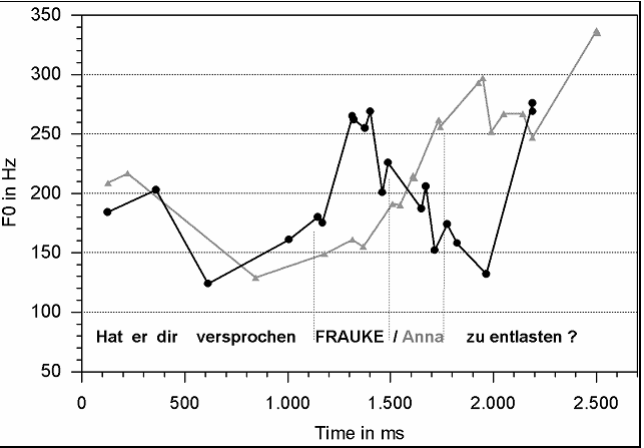
Averaged f0 values of the context questions preceding the target sentences of variant F (black line) and variant G (gray line).

**Figure 2:**
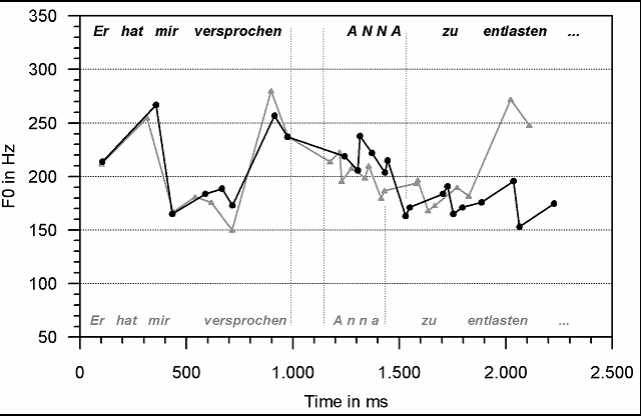
F0 contours of variant F (black line) which comprises the focus accentuation, and condition G (gray line) with the prosodic realization of non-focused given information.

The fundamental frequency pattern (f0) was analyzed for the context questions (F2 and G2, see Table 1), and the target sentences (F3 and G3) per condition using the WinPitch software (Version 1.89; Pitch Instruments Inc.; Toronto, Canada). Figure 1 displays the f0 contour of the context questions (F2 and G2), and Figure 2 illustrates the f0 pattern of the target sentences conveying either a focus accentuation (F3) or the prosody of non-focused given information (G3). Furthermore, duration analyses were conducted for the target sentences. Forty-four realizations per condition entered each averaging procedure. The f0 values were obtained from the speech oscillogram every 20 ms. Zero values (e.g. in speech pauses and unvoiced consonant positions) were treated as missing values. In succession, the onset, the offset, the minimal and the maximal f0 value per segment (phrase-wise and segment-wide) were calculated. Differences in the f0 values and segment durations were analyzed by means of t-tests (two-tailed). In the following sections on fundamental frequency and durational patterns, only the acoustic data for the sentence fragments are reported which are relevant for the ERP analyses.

### Fundamental frequency patterns

In Figure 1, the black line depicts the f0 pattern of the contrastive context question type (question F2 of Table 1) which determines the focus on the noun 'Anna' in the consecutive target sentence. The gray line in Figure 1 visualizes the f0 pattern of the non-contrastive contexts (question G2 of Table 1) preceding the repetition of 'Anna' in the target sentence. It is apparent that the subsequently corrected noun 'Frauke' in the question F2 (black line) bears a prominent rise-fall in the f0, and a subsequently rising question intonation. Question G2, on the other hand, only displays a slowly rising default question intonation without an intermediate prominent f0 peak. Statistical differences between the questions' f0 courses are revealed over their onsets (t [80] = -4.74; p ≤ 01), the noun (t [86] = 10.30; p ≤ 01), and the verb (t [86] = -12.02; p ≤ 01).

Figure 2 illustrates the critical intonation patterns of the target sentence types F3 and G3. The black line displays the f0 course of sentence type F3. In sentence type F3, the noun 'Anna' conveys a focus accent which also influences the realization of surrounding sentence elements. The gray line depicts the f0 course in sentence type G3 with the noun 'Anna' prosodically realized as non-focused given information. It is apparent from Figure 2 that the sentence types F3 (black line) and G3 (gray line) both display a high f0 peak close to the sentence onset. The f0 peak values differ, however, significantly (t [78] = 2.89; p ≤ 01).

Both sentence types further reveal a major prosodic boundary [16] on the verb ('promised'). This boundary is indicated by a pronounced high boundary tone at the verb's last syllable (t [86] = -5.81; p ≤ 01). Furthermore, the verb duration (see consecutive section) and a speech pause after the verb indicate the existence of a major prosodic boundary. Both realizations differ as well in the noun position 'Anna' (t [86] = 3.12; p ≤ 01). Overall, sentence type G3 displays a rather flat falling f0 curve towards the end of the fragment (f0 range= 40 Hz). The f0 contour in sentence type F3 is on the average more pronounced (range= 70 Hz) in noun position and conveys a prominent high peak followed by persistent f0 compression towards the sentence end. In the position of the verb, the f0 excursion between the sentence types F3 and G3 differ in f0 excursion as well (t [86] = -17.69; p ≤ 01) with a stronger pronunciation in type G3.

### Durational properties

In the context questions, the length of the first fragment ('Did he promise you') differs between the question types F2 and G2 (980 ms vs. 1150 ms; t [86]= -5.36; p ≤ 01). Moreover, the duration of the noun differs between realization types with a longer extent of the G2 type (450 ms vs. 530 ms; t [86]= -2.86; p ≤ 01).

In the target sentences ('He promised me') the length of the first fragment of does not differ between the sentence types F3 and G3. The last syllable, however, is longer for sentence type G3 than for F3 (460 ms vs. 420 ms; t [86]= -3.54; p ≤ 01). The pauses after the first fragment do not differ significantly. In the position of the noun focus accent, sentence type F3 bears a longer duration than sentence type G3 (190 ms vs. 150 ms; t [86]= 5.16; p ≤ 01). Moreover, the pause following the noun position 'Anna' is longer in type F3 than in G3 (30 ms vs. 20 ms; t [86]= 2.79; p ≤ 01). The duration of the consecutive verb does not differ between the sentences.

### Procedure

Overall, four conditions (4 × 44 dialogs) were presented to listeners in pseudo-randomized order, and divided into four separate blocks. The interval between the context (F1+F2 or G1+G2) and target sentences (F3 or G3) was 1000 ms. The dialogs were delivered to the listeners via loudspeakers. Participants were instructed about their task. In particular, they had to judge after each dialog whether the prosody of the target sentence matched the preceding context or not by pressing a button on a key-box. Before each target sentence, a fixation cross appeared on the screen and remained until the end of the dialog. By doing so, eye movements of the participants should be avoided. In addition, a visual cue ('Match?') after each dialog served to remind participants of the task.

### ERP recordings

The EEG was recorded in an electromagnetically shielded cabin from 25 Ag/AgCl cap-mounted electrodes. They were placed at FP1, FP2, FZ, F3, F4, F7, F8, FT3, FT4, FT7, FT8, T7, T8, CZ, C3, C4, CP5, CP6, PZ, P3, P4, P7, P8, O1 and O2 following the international 10–20 system [30]. The electrooculogram (EOG) was recorded from electrodes below and above the right eye as well as from the outer canthus of each eye. A ground electrode was placed at the sternum of each participant. On-line, the system was referenced to the left mastoid, and off-line re-referenced to linked mastoids. The EEG and EOG were acquired with PORTI-32 amplifiers and the MREFA software [33] at a sampling frequency of 250 Hz.

### Data analysis

Processing of the EEG data was conducted with the software package EEP 3.2 (Max Planck Institute for Cognitive and Brain Sciences, Leipzig). EEG epochs containing eye blinks or movement artifacts were rejected from the data sets, and did not enter the ERP averages. Averages were computed for 4000 ms after the onset of the target sentences, and for 2000 ms after the onset of the last syllable of the verb 'verspro chen' ('promised') and the verb offset. For all computations, a pre-stimulus baseline of 200 ms was used. First, single subject averages were computed. In succession, means were calculated across subjects. All statistical analyses were conducted on unfiltered ERP data. For the statistical analysis, six lateral ROIs were defined. Each ROI included three electrodes: left anterior (F3, F7, FC3), right anterior (F4, F8, FC4), left central (T7, C3, CP5), right central (T8, C4, CP6), left posterior (P3, P7, O1), right posterior (P4, P8, O2). First, we conducted an overall ANOVA with the factors Context (focus or no focus), Prosody (focus accentuation or prosody of 'given' information), Hemisphere (left or right) and Region (anterior, central or posterior). In addition, we calculated three-way ANOVAs with the factors Condition (matching vs. non-matching combination of context and target sentence), Region (anterior vs. central vs. posterior), and Hemisphere (left vs. right). When interactions of the factor condition with either region or hemisphere were observed, further two-way ANOVAs were computed. When three-way interactions were obtained, further one-way ANOVAs were calculated. All values reported for Condition × Region were adjusted for repeated measurements by means of the Greenhouse-Geisser-Epsilon correction. The time windows (TW) for the statistical analyses consecutively follow the offset of the verb ('versprochen'). They were ad hoc set to a length of 500 ms to enable comparisons of the current data and prior results regarding the CPS in single sentences [12,15]. One TW was analyzed in addition (1100–1600 ms after sentence onset) based on visual inspection of the ERP data.

## Results

### Behavioral results

We first calculated an overall ANOVA over the errors per condition (FF, GG, FG and GF) which were made in judging the appropriateness of the context-accentuation associations. The ANOVA revealed a main effect Condition (F(3,63) = 21.13; p ≤ 01). To explore further on the direction of this main effect, separate t-tests were computed.

As apparent from Table 2, participants were most adequate in judging condition FF (4.7% error rate). In condition FF, the interplay between context and target signals a focus which is then also realized with the appropriate accentuation. The second adequate combination of context and target (condition GG) is yielded errors in 28.8% of the trials. The error rate differs significantly between the conditions FF and GG (t[21] = 3.64; p ≤ 01). With respect to inadequate associations, participants are well able to detect the inappropriate prosody of condition FG as indicated by 9.9% false answers (no statistical difference to condition FF). In condition FG, the target sentence conveys a focus on the noun 'Anna'. However, listeners are confronted with the prosodic pattern of non-focused given information. On the contrary, condition GF yields false answers in 45.7% of the trials. Condition GF provides non-focused given information on the critical noun 'Anna'. However, listeners are presented with the target sentences containing the accentuation of focused information. The error rate for condition GF differs significantly from condition GG (t[21] = -3.07; p ≤ 01) and from condition FG (t[21] = 5.60; p ≤ 01).

**Table 2:** Percentages of erroneous answers per condition

***Context – Target interplay determines***	***Matching Accentuation***	* Non-matching Accentuation
***FOCUS***	Condition FF = 4.7%	* Condition FG = 9.9%
***NO FOCUS (GIVEN)***	Condition GG = 28.8%	* Condition GF = 45.7%

The asterisk (*) signals non-matching combinations of the semantic-pragmatic information structure and the prosodic realization of the target sentence.

### Electrophysiological data

The results of the overall ANOVA revealed a main effect Context in the TW between 0–500 ms (F(1,21)= 4.55; p ≤ 05) and a main effect Prosody in the TW between 1000–1500 ms after verb offset (F(1,21)= 4.77; p ≤ 05).

**Figure 3:**
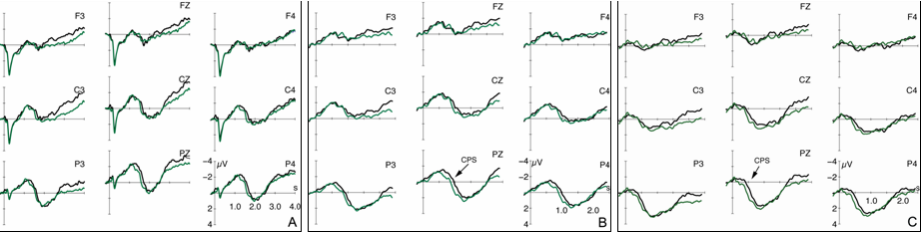
ERPs (5 Hz low-pass filtered) to the target sentences conveying focus accentuation. The black line illustrates the brain responses in matching context (FF), and the green line in non-matching context (GF). ERPs are displayed with varying onsets: *A*. from sentence onset, *B*. from the verb's last syllable, and *C*. noun average starting at verb offset.

**Figure 4:**
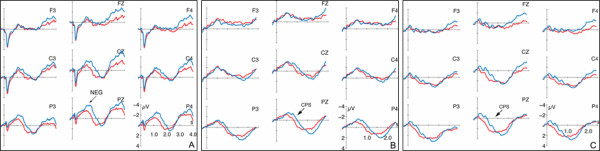
ERPs (5 Hz low-pass filtered) to the target sentences conveying the accentuation of non-focused given information. The red line illustrates the brain responses in matching context (GG), and the blue line in non-matching context (FG). The ERPs are displayed with varying onsets: *A*. from sentence onset, *B*. from the verb's last syllable, and *C*. noun average starting at verb offset.

**Figure 5:**
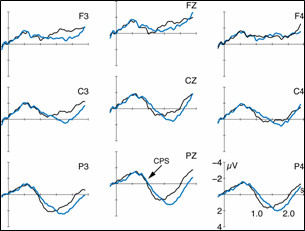
ERPs (5 Hz low-pass filtered) to the target sentences following the 'focus' context (onset is the verb's last syllable – start of prosodic boundary). The black line depicts the brain responses for condition FF which is preceded by a matching context. The blue line shows the deflections for condition FG in which context and accentuation do not match.

**Figure 6:**
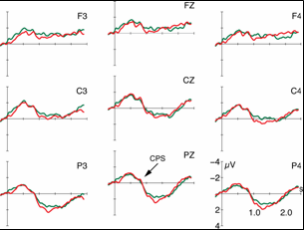
ERPs (5 Hz low-pass filtered) to the target sentences following the 'no focus' context (onset is the verb's last syllable – start of prosodic boundary). The red line depicts the brain responses for condition GG in which context and prosodic realization match, and the green line depicts the ERP for the non-matching condition GF.

The reported ERP data do predominantly convey comparisons between electrophysiological reactions to identical prosodic realizations. By this, confounds of effects due to the semantic-pragmatic structure as opposed to the actual prosodic realization are avoided. First, the comparison of ERPs to condition FF vs. GF are reported, thus for the processing of the focus accentuation (see Figure 3). A second comparison is between the conditions GG and FG. In these conditions, listeners were always presented with the accentuation of non-focused given information (see Figure 4). In addition, a post-hoc statistical analysis was carried out between the dialogs with identical contexts but varying accentuation patterns. It aims at confirming that the ERP onset latencies are identical for the dialogs with identical contexts (FF and FG, GG and GF) and indeed vary as a function of dialog context irrespective of the actual accentuation (see Figure 5 and 6).

### ERPs to the accentuation of focused information

Figure 3 displays the ERP data (5 Hz low-pass filtered only for illustration purposes) to the processing of the target sentences comprising the accentuation of focused information. Thus, only the contexts differ which precede the target sentences with the prosodic realization of the target sentences held constant. In condition FF, the accentuation of the target sentence matches the focus on the noun 'Anna'. In condition GF, on the other hand, the target sentence noun 'Anna' does not convey a focus but non-focused given information. The accentuation is thus inappropriate. Part A of Figure 3 displays the ERPs with an average onset at the beginning of the target sentence, part B with an onset at the prosodic boundary preceding the noun, and part C presents the noun average starting at verb offset. Statistical analyses of the ERPs are based on the part C display. In Figure 3, black lines depict the ERPs to the processing of the accentuation which matches the preceding context (condition FF). The green line illustrates the ERPs for the inappropriate associations (condition GF). In condition GF the interplay of context and target sentence signals non-focused given information on 'Anna' while the sentence bears a focus accentuation.

The noun average (Figure 3C) exhibits a slow centro-posterior positive-going ERP deflection between 100 and 1600 ms for the non-matching condition GF. In the appropriate condition FF, on the other hand, the positivity is delayed. It is apparent between 400 and 1800 ms.

The statistical analyses reveal an interaction of the factors ConditionxRegion in the first considered TW from 0–500 ms (F(2,42) = 15.35; p ≤ 01). Its decomposition attests an effect Condition in the posterior ROI (F(1,21) = 9.91; p ≤ 01). The second TW (500–1000 ms) yields an interaction of the factors Condition × Hemisphere × Region (F(2,42) = 7.30; p ≤ 01). The third TW (1000–1500 ms) reveals interactions of the factors Condition × Hemisphere × Region (F(2,42) = 11.24; p ≤ 01), and of the factors Condition × Hemisphere (F(1,21) = 6.56; p ≤ 05). A three-way interaction is also displayed within the fourth considered TW between 1500–2000 ms (F(2,42) = 4.42; p ≤ 05). Its decomposition reveals effects Condition in the left anterior(F(1,21) = 7,08; p ≤ 05), the right central (F(1,21) = 6,75; p ≤ 05), and the right posterior ROI (F(1,21) = 5,62; p ≤ 05).

### ERPs to the prosody of non-focused given information

Figure 4 displays the ERP data (5 Hz low-pass filtered for illustration purposes) to the processing of the target sentences comprising the prosodic pattern of non-focused given information. Once more, the ERPs to prosodically identical realizations are reported which vary in the contexts preceding them. In condition GG, the accentuation of the target sentence matches the pragmatic structure of the dialog. However, in condition FG the interplay between context and target sentence induces a focus in the noun position 'Anna' which is not reflected in an adequate focus accentuation. In Figure 4, red lines depict the ERPs to the processing of condition GG. The blue line illustrates the ERPs to condition FG.

As in Figure 3, part A of Figure 4 displays the ERPs with an average onset at the beginning of the target sentence, part B with an average starting at the prosodic boundary preceding the noun, and part C displays the noun average starting at verb offset.

The statistical analyses of the positive-going ERPs were computed in successive time windows of 500 ms based on part C of Figure 4. As apparent, condition GG exhibits a centro-posterior positive ERP between 100 and 1300 ms. For the inappropriate condition FG, on the contrary, the positivity is delayed and apparent between 400 and 1500 ms.

The statistical analyses prove a main effect Condition in the first considered TW between 0–500 ms (F(1,21) = 9.35; p ≤ 01). An additional interaction Condition × Region (F(2,42) = 5.54; p ≤ 05) in this TW indicates the scope of the effect Condition especially to the central (F(1,21) = 7,85; p ≤ 05), and the posterior ROI (F(1,21) = 17,10; p ≤ 01). The second TW (1000–1500 ms) exclusively yields interactions of the factors Condition × Hemisphere × Region (F(2,42) = 5.03; p ≤ 05), and the factors Condition × Region (F(2,42) = 6.65; p ≤ 05). No effects are apparent in the third TW. The fourth computed TW (1500–2000 ms) again reveals an interaction of the factors Condition × Region (F(2,42) = 8.23; p ≤ 01). Its decomposition attests an effect Condition in the anterior ROI (F(1,21) = 4,39; p ≤ 05).

In addition, visual inspection of the Figure 4A reveals the existence of a centro-posterior negative-going ERP for condition FG which is most prominent between 1100–1600 ms post target sentence onset. The ANOVA for this TW yields an interaction of the factors Condition × Region (F(2,42)= 9.90; p ≤ 01) whose decomposition manifests an effect Condition in the posterior ROI (F1,21)= 14.56; p ≤ 01).

### Contextual dependence of the ERP onset latencies

As apparent from part B and C of the Figures 3 and 4, the varying onset latencies between conditions do not rely on the chosen ERP average onset positions (part B= verb's last syllable; part C= verb offset for noun average). Rather, visual inspection reveals that the onset latencies of the positive ERP deflections to the conditions GG and GF are approx. 300 ms earlier than the positivities to the conditions FF and FG. This difference cannot be accounted for by the pause accompanying the prosodic boundary before the noun as the pause does not differ between conditions. In Figure 5 and 6, the conditions are thus plotted for illustration reasons by means of identical contexts but varying accentuation patterns.

Figure 5 displays the average for the conditions FF (black line) and FG (blue line) starting the verb's last syllable. In both conditions, the differing prosodic realizations are this time preceded by the context question which establishes a focus on the noun 'Anna' of the target sentence. Condition FF and FG display similar onset latencies for the positive-going ERP at approx. 800 ms after the onset of the prosodic boundary. A post-hoc statistical analysis in TW of 500 ms was conducted for all electrodes from the onset of the prosodic boundary. These earlier average onsets were chosen for the post-hoc analysis due to the previously found noun-preceding triggers of the CPS (i.e. effects Condition already present in the TW 0–500 ms in the noun onset average) in those conditions with contexts establishing 'given' information in the target sentence (i.e. condition GF in Figure 3, and condition GG in Figure 4). No statistical differences could be proven.

Figure 6 shows the ERPs for the conditions GG (red line) and GF (green line). Here, the target sentences are preceded by the context question in which the noun 'Anna' is mentioned for the first time and which bears a default question intonation. In turn, the target sentences of both conditions do not convey a noun focus. Condition GG and GF again display similar onset latencies for the positive-going ERP but this time approx. 500 ms after the onset of the prosodic boundary. The post-hoc statistical analysis uncovers an effect Condition in the initial TW between 0–500 ms after prosodic boundary onset (F(1,21)= 8.94; p ≤ 01). However, no effect is present in the successive TW which is convergent with the onset of the positive ERP.

## Discussion

The present study was conducted to investigate the electrophysiological responses to spoken dialog perception. In particular, the study aimed at delineating the influence of contextual-pragmatic and prosodic information on the structuring of quasi-natural connected speech. For this purpose, listeners were presented with dialogs containing focused contrastive (conditions FF and FG) vs. non-focused given information in the target utterances (conditions GG and GF). Moreover, the dialogs either comprised an adequate (FF and GG) or an inadequate accentuation (condition FG and GF) with respect to the semantic-pragmatic focus.

The behavioral results indicate that listeners are not always certain which prosody should accompany a certain information structure. In fact, the judgment task seems to be easier when the target sentences convey focused information irrespective of whether it is realized with an appropriate (condition FF) or an inappropriate accentuation (condition FG). These outcomes resemble prior behavioral results on listeners' identification of mis-realized focus accentuation [7,31]. However, the evaluation of accentuation patterns is much easier for listeners when the 'under'-accentuation of focused information is encountered (condition FG) in contrast to an 'over'-accentuation of given information (condition GF).

In general, listeners seem less aware of the accentuation which appends to non-focused given information (condition GG and GF). With respect to condition GF, this finding is again in congruence with prior findings [5,7,31]. Alternatively, participants' inaccuracy in condition GG could also be attributed to an additional facet of communication. It is rather unusual for interlocutors to repeat statements just made by someone else (apart from showing surprise about it which would then require a particular intonation). Rather, speakers signal approval of an interlocutor's statement by uttering 'Yes' or 'That's right'. We do assume that the behavioral responses to condition GG and GF are at least partly attributable to the violation of cooperation principles in conversation [32] as information from the context is completely repeated in the target sentences.

The ERP data in general show a centro-posterior positive deflection for all conditions. However, the positive shift varies in onset latency. As apparent from the Figures 3 and 4, the positivities neither diverge as a function of the prosodic realization of the target utterances nor the ERP average onset. Figure 3 only comprises ERPs to the focus accentuation variant of the dialogs, and Figure 4 only the responses to the accentuation of non-focused given information. Moreover, the difference between conditions cannot be ascribed to the actual ERP average chosen (B: verb syllable onset; C: noun average starting from verb offset). The onset latency of the positive shifts yet differs as a function of the contexts preceding the target sentences. An effect Context in the time window from 0–500 ms of the noun average statistically corroborates the descriptive difference.

As further apparent from Figures 5 and 6, the conditions with identical contexts preceding the target sentences result in similar latencies of the positive going ERP component. In those target sentences which are preceded by a 'focus' question (condition FF and FG) the positivity starts ~300 ms later than in those targets which are preceded by a 'no focus' question. While the conditions GG and GF do both not convey a noun focus in the target, the conditions FF and FG comprise of such a focus position.

Thus, the information structural interplay of context and target sentences seems to direct the structuring of spoken dialogs. In particular, the focus structure of a dialog predominantly influences the interpretation of the dialogic information irrespective of the actual accentuation of the target.

In both 'no focus' conditions (GG and GF) the centro-posterior positivity appears with a temporal lag of ~500 ms to the onset of the prosodic boundary on the verb. Thus, it strongly resembles the Closure Positive Shift (CPS) known from single sentence processing [12,15] as a marker of online speech structuring.

Crucially, listeners make use of the prosodic boundary cues for utterance structuring before encountering the noun position when processing condition GG and GF. This strategy can only be attributed to listeners' exploitation of the context cues, namely the question intonation. In both 'no focus' conditions (GG and GF) the contexts are accompanied by default question prosodies. We propose that these contextual cues together with the acoustic event of a prosodic boundary in the target sentence then lead listeners towards the utilization of the boundary for utterance structuring. In turn, a CPS is elicited when listeners perceive the prosodic boundary on the verb.

In both 'focus' conditions (FF and FG), on the other hand, the positive shift is apparent with a latency of ~500 ms after the focused noun has been encountered, i.e. ~300 ms later that in the 'no focus' conditions. Due to the scalp topography, the latency and the morphology of the positive-going ERP in the conditions FF and FG we also interpret the deflection as CPS. In contrast to the 'no focus' conditions GG and GF, however, the CPS in the 'focus' conditions (FF and FG) is induced by the processing of the noun focus in the target sentences.

In accordance with prior research [18], our findings suggest that the structuring of spoken language manifests in a similar ERP component, the Closure Positive Shift (CPS). In contrast to the CPS elicited by context-free sentence presentation, however, the events which induce the component during the perception of context-embedded utterances (i.e. dialogs) differ. When conversational contexts lead listeners to anticipate an information center or focus, respectively, they use the focus position to structure the utterance. On the other hand, when the context of an utterance do not guide listeners towards the expectation of an information center, they use major prosodic boundaries for structuring as they also do by default when perceiving context-free single sentences [12,14,15].

Further, our results show that the latency of the CPS does not vary as a function of the appropriateness in the accentuation of the target sentence with respect to a preceding context. Under the 'no focus' context, similar latencies are yielded for the conditions with the appropriate accentuation (GG) and the inappropriate accentuation (GF); under the 'focus' context alike CPS timing is apparent for the conditions with the appropriate accentuation (FF) and the inappropriate accentuation (FG). Thus, our study can complement the results of Hruska and coworkers [18] in showing that the elicitation of the CPS during dialog perception predominantly relies on contextual factors irrespective of the actual accentuation of a dialog's target.

Apart from the finding of a contextual dependence of the CPS in dialog perception, however, there is some indication that listeners can perceive a contextually inadequate accentuation, too. However, this effect is only present when a dialog context signals a focus in the target sentence which is then prosodically realized as non-focused given information (condition FG, see Figure 4A). The inappropriate 'under' accentuation then evokes a sustained centro-posterior negative deflection (NEG) which is statistically reliable from 1100–1600 ms after the onset of the target sentence.

According to the prosodic analyses, the descriptive onset of the negativity precedes the onset of the focused but unaccented noun. Thus, listeners must be readily able to exploit the subtle prosodic cues conveyed by the sentence-initial fragment ('He promised me'). The prosodic inadequacy of the target sentence emerges further and reaches statistical significance when the absence of the focus accent on the noun is detected.

Similar negative deflections in dialog comprehension have been reported by Hruska et al. [17,18] for German, and Magne et al. [23] for French. These negativities were interpreted as N400 responses due to integration problems of focused but unaccented words into the information structure of a dialog. Within the current design with quasi-natural connected speech, however, the onset of the negative ERP component can hardly be fixed on to one discrete element of the target sentence. Apparently, the negative deflection in our study does also not impede the context-bound occurrence of the CPS. In terms of scalp topography and eliciting factors, the negativity (NEG) for condition FG in our study coincides with the previously reported N400 for missing focus accents [17,23]. Moreover, it resembles N400 reports on discourse-bound semantic processing [24,25]. With our present materials and design, however, we cannot make unequivocal assertions as to the timing of the NEG component.

In addition to the negativity elicited during the processing of condition FG, the effect Prosody in the time window from 1000–1500 ms of the overall ANOVA (verb offset average) also indicates an additional process accompanying the CPS. Although the ERPs in condition FG show a more pronounced positive deflection than in condition GG (cf. Figure 4C), no effect Condition was yielded within this time window from 1000–1500 ms. Such a P600-like effect could further corroborate the interpretation of the negativity in our study as an N400. In particular, it might indicate that a missing focus accent not only causes meaning-related integration problems but also hinders the constitution of the information structure of a dialog. Further exploration on this issue would, however, require experimental manipulations at the cost of the naturalness of the dialog situations. Either, the utterance-internal prosodies would have to be made artificially identical or only the processing of sentence-initial focus positions could be explored. Moreover, previous studies [20,22] have shown that the perceptual consequences of sentence-initial vs. sentence-medial accents are hardly comparable, even manifesting in reversed polarities in the ERPs.

## Conclusion

The current study provides a first link between the perceptual events listeners use when structuring spoken language in the absence vs. presence of context information. The data indicate that contextually embedded utterances (dialogs) which do not enhance the information status of interlocutors by information foci are structured in a similar fashion than context-free single sentences. The online segmentation process is then guided by the major prosodic boundaries implicit to the speech signal. In contrast, dialogs which add to the information status of an interlocutor are structured online by means of the information center or focus, respectively, as it alters the current state of knowledge of a listener. The differing and rather eclectic processing strategies only attribute a minor role to the final speaker-dependent accentuation of the conveyed message. Listeners strongly consider the context in which their communication partners have made a particular statement.

## Competing interests

The author(s) declare that they have no competing interests.

## Authors' contributions

UT designed and analyzed the materials for the experiment, programmed it, collected a majority of the data, analyzed the data, and drafted the initial paper. AP provided support on the statistics and for manuscript preparation and revision. KA, the head of the research project, contributed to the initial conception of the study and provided consultation for the experiment and manuscript preparation. All authors have read and approved the final manuscript.
